# Мелатониновый статус у пациенток с ожирением и дисфункцией яичников в репродуктивном возрасте

**DOI:** 10.14341/probl12849

**Published:** 2022-01-27

**Authors:** Е. Н. Андреева, О. Р. Григорян, Ю. С. Абсатарова, Е. В. Шереметьева, Р. К. Михеев

**Affiliations:** Национальный медицинский исследовательский центр эндокринологии; Московский государственный медико-стоматологический университет им. А.И. Евдокимова; Национальный медицинский исследовательский центр эндокринологии; Национальный медицинский исследовательский центр эндокринологии; Национальный медицинский исследовательский центр эндокринологии; Национальный медицинский исследовательский центр эндокринологии

**Keywords:** мелатонин, ожирение, менструальная дисфункция, нарушения сна

## Abstract

**ОБОСНОВАНИЕ:**

ОБОСНОВАНИЕ. Мелатонин – главный гормон эпифиза. Регулируя циркадианные ритмы,  являясь иммуннорегулятором и антиоксидантом, данный гормон принимает участие в работе яичников: его высокие концентрации блокируют апоптоз и нейтрализуют активные формы кислорода, участвующие в фолликулогенезе, овуляции, созревании яйцеклетки и образовании желтого тела.

**ЦЕЛЬ:**

ЦЕЛЬ. Изучить мелатониновый статус и его взаимосвязь с менструальной дисфункцией и нарушениями сна у пациенток репродуктивного возраста с ожирением.

**МАТЕРИАЛЫ И МЕТОДЫ:**

МАТЕРИАЛЫ И МЕТОДЫ. В одномоментном сравнительном исследовании приняли участие женщины 18-35 лет: 30 пациенток с ожирением и нарушениями менструальной функции неорганического характера и 30 здоровых женщин группы сравнения с нормальным весом и регулярным менструальным циклом. Все участницы прошли анкетирование для выявления сомнологических нарушений, а также был исследован уровень мелатонина в слюне и 6-сульфатоксимелатонина в моче.

**РЕЗУЛЬТАТЫ:**

РЕЗУЛЬТАТЫ. В группе пациенток с ожирением (n=30) в 47% случаев встречались различные нарушения сна (р=0,003), в том числе чаще регистрировали синдром обструктивного апноэ сна (30% случаев), а также была обнаружена корреляция между показателями анкетирования субъективных характеристик сна и индексом массы тела пациенток (r=0,450, p=0,030) по сравнению с группой здоровых женщин с нормальным весом (n=30). В основной группе уровень мелатонина в слюне оказался статистически значимо ниже по сравнению с контролем: медиана 12,6 пг/мл и 25,5 пг/мл, соответственно (р=0,008), та же закономерность была зарегистрирована для 6-сульфатоксимелатонина: 14,72 пг/мл и 31,12 пг/мл, соответственно.

**ЗАКЛЮЧЕНИЕ:**

ЗАКЛЮЧЕНИЕ. Пациентки с ожирением и менструальной дисфункцией чаще страдают различными нарушениями сна и имеют более низкие показатели мелатонина в слюне и 6-сульфатоксимелатонина в моче.

## Abstract (en)

**BACKGROUND:**

BACKGROUND: Melatonin is the main hormone of the pineal gland. By regulating circadian rhythms and being an immune regulator and antioxidant, this hormone takes part in the work of the ovaries: its high concentrations block apoptosis and neutralize reactive oxygen species involved in folliculogenesis, ovulation, egg maturation and corpus luteum formation.

**AIM:**

AIM: To study melatonin status and its relationship with menstrual dysfunction and sleep disorders in obese women of reproductive age.

**MATERIALS AND METHODS:**

MATERIALS AND METHODS. In a one-stage comparative study, women 18-35 years old took part: 30 patients with obesity and menstrual disorders of an inorganic nature and 30 healthy women in the comparison group with normal weight and regular menstrual cycle. All participants underwent a questionnaire to identify somnological disorders, and the level of melatonin in saliva and 6-sulfatoxymelatonin in urine was also investigated.

**RESULTS:**

RESULTS: In the group of patients with obesity (n=30), various sleep disorders were encountered in 47% of cases (p=0.003), including more often obstructive sleep apnea syndrome was recorded (30% of cases), and a correlation was found between the indicators of the questionnaire survey of subjective sleep characteristics and body mass index of patients (r=0.450, p=0.030) compared with a group of healthy women with normal weight (n=30). In the main group, the level of melatonin in saliva was statistically significantly lower than in the control: median 12.6 pg / ml and 25.5 pg / ml, respectively (p=0.008), the same pattern was recorded for 6-sulfatoxymelatonin: 14, 72 pg / ml and 31.12 pg / ml, respectively.

**CONCLUSION:**

CONCLUSION: Patients with obesity and menstrual dysfunction are more likely to suffer from various sleep disorders and have lower levels of melatonin in saliva and 6-sulfatoxymelatonin in urine.

## ОБОСНОВАНИЕ

Мелатонин — гормон, который в основном синтезируется в ткани эпифиза в ночное время и контролирует циркадианные ритмы млекопитающих [1]. Он также вырабатывается в других тканях и органах: кишечнике, яичниках, сетчатке и других [2]. Помимо регулирующей циркадианные ритмы функции, мелатонин обладает антиоксидантными, противовоспалительными, антиапоптотическими и регенерирующими свойствами. Исследования показывают, что данный гормон влияет на функцию жировой ткани, метаболизм липидов и массу тела [3].

Ожирение — одна из самых распространенных медицинских проблем в развитых странах во всех возрастных группах, среди и мужчин, и женщин [4]. Рост распространенности избыточной массы тела и ожирения — серьезная угроза общественному здоровью во всем мире. В последнее время внимание исследователей привлекает возможная роль мелатонина в регуляции массы тела и предотвращении метаболических нарушений, связанных с ожирением.

Механизм регулирующего влияния мелатонина на массу тела довольно сложен. Во-первых, гормон контролирует дифференцировку адипоцитов и адипогенез [5]. Во-вторых, мелатонин индуцирует образование «бежевых» адипоцитов и потемнение белой жировой ткани, увеличивая термогенную способность и расход энергии, препятствуя тем самым увеличению массы тела [6]. В-третьих, мелатонин способствует повышенному образованию бурой жировой ткани и активации ее функции [7]. Еще один возможный механизм связывают с изменением микробиоты кишечника: на экспериментальных моделях на фоне дотации экзогенного мелатонина было продемонстрировано преобладание микробных сообществ, ассоциированных с позитивными изменениями липидного и энергетического метаболизма [8]. Следует учитывать также антиоксидантные и противовоспалительные свойства гормона, который потенцирует снижение массы тела, устраняя дисфункцию адипоцитов и митохондрий [9]. Дополнительный прием мелатонина восстанавливал секрецию и метаболизм адипонектина у мышей с ожирением, что тоже может объяснить его защитный эффект в отношении увеличения массы тела [4].

Регуляторный механизм действия мелатонина варьирует в зависимости от органа-мишени. В поджелудочной железе гормон снижает уровни циклического аденозинмонофосфата (цАМФ) и циклического гуанозинмонофосфата (цГМФ), через которые осуществляется контроль за гомеостазом инсулина и глюкозы [10]. В результате этого процесса происходят ритмические колебания чувствительности тканей к глюкозе, максимальной в утренние часы и минимальной ночью [11]. Что касается других физиологических эффектов, то антиоксидантная способность мелатонина в основном связана с увеличением производства антиоксидантных ферментов, включая супероксиддисмутазу, каталазу, глутатион, а также с его антиоксидантным действием как таковым [12].

Известно, что депривация сна — один из факторов риска развития ожирения [4]. Эпидемиологические исследования выявили взаимосвязь между хроническим недосыпанием и увеличением массы тела [13]. Циркадианные ритмы являются фундаментальными биологическими процессами, которые регулирует цикл «день-ночь», обеспечивая периодичность биохимических, молекулярных и физиологических процессов. Появляется все больше доказательств связи системы биологических часов, метаболизма и энергетического баланса [13]. Изменения в образе жизни, такие как увеличение времени бодрствования, использование искусственного света, телевидения, компьютерных мониторов и других средств массовой информации, нарушают циркадианные ритмы. Одним из последствий является нарушение режима секреции мелатонина. В клинических исследованиях была обнаружена обратная связь между уровнем мелатонина и степенью ожирения [4]. Снижение уровня гормона усугубляет инсулинорезистентность и выраженность метаболического синдрома [14].

Регулируя циркадианные ритмы и являясь антиоксидантом, мелатонин, в том числе, принимает участие в работе яичников: его высокие концентрации блокируют апоптоз ооцитов и нейтрализуют активные формы кислорода, участвующие в фолликулогенезе, овуляции, созревании яйцеклетки и образовании желтого тела. Данный гормон является одним из ключевых нейроэндокринных регуляторов работы оси гипоталамус-эпифиз-гонады, влияя на синтез и секрецию гонадотропинов [15]. Было показано, что мелатонин увеличивает экспрессию белков кисспептинов, которые являются важными регуляторными молекулами в секреции лютеинизирующего и фолликулостимулирующего гормонов [16][17]. Положительный эффект «гормона сна» на фертильность, развитие ооцитов и поддержание эмбриона объясняется его способностью уменьшать окислительное повреждение фолликулов яичников. В то же время более высокие концентрации мелатонина, наблюдаемые во время беременности, по-видимому, поддерживают созревание плода и гомеостаз плаценты [18].

Одним из частых коморбидных состояний у пациенток с ожирением является менструальная дисфункция. Сопутствующий дефицит мелатонина у женщин с повышенным индексом массы тела (ИМТ) может непосредственно усугублять нарушения менструального цикла. Антиоксидантные свойства мелатонина и его влияние на ось гипоталамус-эпифиз-яичники способствуют уменьшению окислительного повреждения фолликулов, улучшая продукцию прогестерона в лютеиновой фазе цикла и созревание ооцитов [19]. Циркулирующий мелатонин может поглощаться яичником, при этом фолликул сам обладает способностью синтезировать и секретировать собственный гормон, тем самым демонстрируя его важную паракринную роль в женской репродуктивной системе [20]. На экспериментальных моделях было продемонстрировано, что после пинеалэктомии развивается овуляторная дисфункция, а добавление экзогенного мелатонина в питание в течение 12 мес улучшает количество и качество ооцитов, а также увеличивает размер помета [21][22].

В клиническом исследовании на группе пациенток с синдромом поликистозных яичников, который часто сопровождается метаболическими и репродуктивными нарушениями, был продемонстрирован положительный эффект дотации экзогенного мелатонина на менструальную функцию и андрогенный профиль [23]. Антиандрогенный потенциал нейрогормона подтверждает его способность снижать уровень цАМФ в гранулезных клетках, что в конечном итоге приводит к уменьшению выработки мужских гормонов [15].

Косвенно оценить уровень мелатонина в крови позволяет измерение его уровня в слюне и его метаболита 6-сульфатоксимелатонина в моче, при этом ИМТ может оказывать значимое влияние на вышеуказанные параметры [24].

## ЦЕЛЬ ИССЛЕДОВАНИЯ

Изучить мелатониновый статус и его взаимосвязь с менструальной дисфункцией и нарушениями сна у пациенток репродуктивного возраста с ожирением.

## МАТЕРИАЛЫ И МЕТОДЫ

Место проведения. Исследование было выполнено на базе ФГБУ «НМИЦ эндокринологии» Минздрава России. Время исследования. Июль 2020 г. — апрель 2021 г.

Изучаемые популяции: 2 популяции (пациентки с ожирением и группа контроля — здоровые женщины).

Популяция «пациентки с ожирением»

Критерии включения: женщины 18–35 лет; ИМТ ≥30 кг/м2; нарушения менструального цикла неорганического характера; отсутствие любой гормональной терапии или препаратами мелатонина в течение 6 мес перед исследованием; получение информированного согласия на комплексное обследование.

Критерии исключения: беременность, лактация; наличие хронических экстрагенитальных заболеваний в стадии обострения, субкомпенсации; наличие декомпенсированной эндокринной патологии; выявление острых и обострений хронических воспалительных заболеваний половых органов; курение, злоупотребление алкоголем.

Популяция «здоровые женщины»

Критерии включения: ИМТ 19,5–25,0 кг/м2, наличие регулярного менструального цикла; отсутствие какой-либо гормональной терапии или препаратами мелатонина в течение 6 мес до исследования, получение информированного согласия на комплексное обследование.

Критерии исключения: наличие хронических экстрагенитальных заболеваний в стадии обострения, субкомпенсации; наличие декомпенсированной эндокринной патологии; выявление острых и обострений хронических воспалительных заболеваний половых органов; курение, злоупотребление алкоголем.

Выборка была сформирована путем сплошного включения наблюдений.

Дизайн исследования: одноцентровое одномоментное сравнительное исследование.

Описание медицинского вмешательства. Проведение клинико-лабораторного обследования: анкетирование (анкета балльной оценки субъективных характеристик сна, анкета скрининга синдрома апноэ во сне, Эпвортская шкала оценки дневной сонливости), гормональное исследование (определение уровня мелатонина в слюне в 3 ч ночи, определение уровня метаболита мелатонина 6-сульфатоксимелатонина в суточной моче).

Методы

В основную группу входили пациентки с различными нарушениями менструального цикла (частые — менее 21 дня, редкие менструации — более 35 дней, аменорея) и ИМТ≥30 кг/м2. В группу сравнения были включены здоровые женщины с регулярным менструальным циклом продолжительностью от 21 до 35 дней и ИМТ в пределах 19,5–25 кг/м2. Все участницы прошли анкетирование с помощью анкеты балльной оценки субъективных характеристик сна (оценка продолжительности сна, времени засыпания, количества сновидений и ночных пробуждений, качества утреннего пробуждения). Показатель более 22 баллов говорил об отсутствии нарушений, пограничные значения колебались от 19 до 21 баллов, значение менее 19 баллов расценивали как расстройства сна [25]. Анкета скрининга синдрома апноэ во сне использовалась для оценки риска данного состояния: при показателе более 4 баллов диагностировали синдром обструктивного апноэ. Для оценки дневной сонливости применяли Эпвортскую шкалу (в норме показатель менее или равен 9 баллам).

Все участницы осуществляли самостоятельный сбор слюны дома в специальные контейнеры со стерильным тампоном в 3 ч ночи при отсутствии или минимальном освещении (слабый ночник). Тампон необходимо было поместить в ротовую полость, где он должен был находиться в течение 3 мин, далее без помощи рук его надо было поместить обратно в пробирку. Емкость можно было хранить в обычном холодильнике при температуре не выше +6°С. Контейнер доставляли на следующий день для исследования методом ELISA.

Сбор суточной мочи для определения 6-сульфатоксимелатонина методом ELISA осуществлялся в течение дня, начиная со второй порции после ночного сна в течение следующих суток. Емкость для сбора мочи хранилась в обычном холодильнике при температуре не выше +6°С. Для исследования была использована порция мочи 20 мл, в том числе учитывался общий суточный объем.

Статистический анализ

Статистическая обработка полученных результатов проводилась с помощью прикладных программ STATISTICA (StatSoft Inc. США, версия 6.0). Объем исследуемых групп был выбран с учетом проводимых ранее исследований аналогичного дизайна, а также положения о минимальном числе участников — 30 человек в одной исследуемой подгруппе. В связи с наличием небольших объемов выборки и распределения, отличающегося от нормального, использовались непараметрические методы статистики. U-критерий Манна–Уитни применяли для сравнения независимых групп по количественным признакам. Анализ таблиц сопряженности с использованием двустороннего точного критерия Фишера, а также критерия χ2 для несвязанных групп использовали для сравнения независимых групп по качественным признакам. Непараметрический метод ранговой корреляции по Спирмену применяли для анализа корреляции двух количественных признаков, а количественного и порядкового — по Кендаллу. Статистически значимыми считали различия при p<0,05.

Этическая экспертиза

Протокол исследования был одобрен этическим комитетом ФГБУ «НМИЦ эндокринологии» Минздрава России (№11 от 22.07.2020).

## РЕЗУЛЬТАТЫ

Всего в исследовании приняли участие 60 женщин: 30 пациенток основной группы и 30 женщин группы сравнения (здоровые участницы). Пациентки были сопоставимы по возрасту и статистически значимо отличались по ИМТ (табл. 1).

При анализе данных анкет балльной оценки субъективных характеристик сна женщины с ожирением и нарушениями менструального цикла статистически значимо отличались от группы контроля по уровню патологических отклонений (p=0,003) и чаще страдали сомнологическими нарушениями (табл. 2).

Мы обнаружили статистически значимую прямую корреляцию между количеством баллов согласно анкете балльной оценки субъективных характеристик сна и ИМТ в основной группе: чем выше был ИМТ, тем хуже были показатели сомнологического профиля по данным анкетирования (r=0,450; p=0,030, метод ранговой корреляции по Кендаллу).

По данным опросника, синдром обструктивного апноэ был статистически значимо более вероятен у пациенток с ожирением, чем в контрольной группе, хотя по уровню сонливости участницы были сопоставимы (табл. 3).

Анализ показателей мелатонина в слюне и его метаболита в суточной моче показал, что пациентки с ожирением и нарушениями менструального цикла имели более низкие значения изучаемых параметров по сравнению с группой здоровых женщин (табл. 4).

**Table:** Таблица 1. Распределение участниц исследования по индексу массы тела и возрасту

Показатель	Основная группа, n=30	Группа контроля, n=30	p (U-тест)
ИМТ, кг/м2	33,8 [ 30,6; 40,1]	20,9 [ 20,0; 22,8]	0,001
Возраст, лет	25 [ 21; 34]	26 [ 22; 35]	0,865

**Table:** Таблица 2. Частота отклонений сна в группе ожирения и группе контроля (p=0,003, критерий χ2)

Параметры сна	Основная группа, n=30	Группа контроля, n=30
Сон нарушен, n (%)	14 (46,7%)	5 (16,7%)
Нормальный сон+пограничный результат, n (%)	16 (53,3%)	25 (83,3%)

**Table:** Таблица 3. Сомнологические характеристики участниц исследования

Показатель	Основная группа, n=30	Группа контроля, n=30	р (критерий Фишера)
Апноэ во сне, n (%)	9 (30)	1 (3)	0,006
Наличие сонливости, n (%)	12 (40)	10 (33)	0,081

**Table:** Таблица 4. Мелатониновый статус участниц исследования

Показатель	Основная группа, n=30 Медиана, 25–75% процентили	Группа контроля, n=30 Медиана, 25–75% процентили	p (U-тест)
6-сульфатоксимелатонин в суточной моче, мкг/24 ч	14,72 [ 13,08; 17,35]	31,12 [ 20,43; 46,82]	0,008
Мелатонин в слюне в 3 ч ночи, пг/мл	12,6 [ 5,99; 23,40]	25,5 [ 8,60; 32,56]	0,003

При корреляционном анализе была выявлена статистически значимая прямая корреляция уровня 6-сульфатоксимелатонина в суточной моче и мелатонина в слюне в 03.00 ночи (r=0,850; р=0,020, метод ранговой корреляции по Спирмену), а также уровня мелатонина в слюне и степени нарушения сна по данным анкеты балльной оценки субъективных характеристик сна (r=0,640; р=0,03, метод ранговой корреляции по Спирмену).

## ОБСУЖДЕНИЕ

Ожирение и его осложнения представляют собой серьезную проблему во всем мире. В последние десятилетия механизмы, лежащие в основе прогрессирования ожирения, активно изучаются. Предрасполагающими к развитию заболевания факторами являются поведенческие (высококалорийная диета, низкая физическая активность и недосыпание) и внутренние (дисрегуляция жировой ткани, хроническое воспаление, окислительный стресс) [26]. В научном сообществе активно изучается возможная связь между дефицитом мелатонина и ожирением. В некоторых работах были продемонстрированы многообещающие результаты относительно потенциальной роли мелатонина в профилактике ожирения и его осложнений [4].

В нашем исследовании был проведен анализ показателей мелатонинового обмена (мелатонин в слюне и 6-сульфатоксимелатонин в суточной моче) в зависимости от наличия ожирения и хронобиологических нарушений у пациенток с менструальной дисфункцией, а также у здоровых женщин группы контроля. Был изучен сомнологический профиль участниц, а также взаимосвязь этих параметров с уровнем мелатонина в моче и слюне. По данным исследования, пациентки основной группы (ожирение) чаще страдали сомнологическими нарушениями, в том числе синдромом обструктивного апноэ. Нами была обнаружена корреляция между нарушениями сна и ИМТ у женщин с нарушениями менструальной функции. Предположительно, как следствие сомнологических расстройств участницы 1-й группы имели пониженное содержание мелатонина в слюне и 6-сульфатоксимелатонина в суточной моче. В том числе, была обнаружена корреляция содержания гормона в слюне и степени нарушений сна по данным анкеты балльной оценки субъективных характеристик сна, что тоже может подтверждать влияние депривации сна на выработку нейрогормона в эпифизе. Данные закономерности были зарегистрированы и в работах других авторов. Согласно результатам D. Corbalán-Tutau и соавт., у женщин с метаболическим синдромом наблюдалась сниженная амплитуда суточного ритма мелатонина. Было установлено, что циркадианный ритм гормона имел обратную корреляцию с уровнями лептина в плазме – одним из основных метаболических гормонов [27].

R. Reiter и соавт. была обнаружена связь между депривацией сна и нарушенной секрецией мелатонина [28]. Интересно, что короткая продолжительность сна коррелировала со снижением уровня лептина и повышенными значениями ИМТ в когортном исследовании, проведенном S. Taheri и соавт. [29]. В работе R. Markwald и соавт. 5 дней недостаточного сна в группе из 16 здоровых взрослых добровольцев привели к энергетическому дисбалансу, нарушенному циркадианному ритму мелатонина и повышению уровня лептина [13].

Короткую продолжительность сна, определяемую как сон 6 ч или меньше за 24-часовой период времени, имеют треть взрослого населения [30]. Нарушения сна чаще встречаются у женщин, чем у мужчин, и часто совпадают с колебаниями половых гормонов [31]. Связь нарушенных циркадианных ритмов и менструальной дисфункции была зарегистрирована в исследованиях с участием женщин, работающих посменно или в ночное время. Участницы, имевшие вышеуказанный график работы, имели различные отклонения в длительности, обильности менструаций или продолжительности менструального цикла [32]. В недавнем проспективном исследовании, куда были включены 287 недавно нанятых на работу медсестер с посменной работой, были изучены показатели качества сна в дополнение к количественной оценке сменного графика работы. Нарушение менструального цикла определялось как наличие цикла менее 21 дня или более 35 дней по крайней мере один раз в течение 6-месячного периода наблюдения. Бессонница была связана с 2-кратным увеличением вероятности нарушений менструального цикла, а нерегулярность цикла увеличивалась по мере нарастания тяжести бессонницы [33].

Относительно небольшое количество эпидемиологических исследований изучали нарушения сна и фертильность женщин. В работе с участием 22 пациенток, перенесших экстракорпоральное оплодотворение (ЭКО), измерялись продолжительность и качество сна в течение одного цикла ЭКО с использованием актиграфа и ежедневных дневников [34]. Исследователи обнаружили линейную связь между общим временем сна и количеством извлеченных ооцитов. В исследование I.D. Wang и соавт. были включены пациентки из национального регистра: 16 718 женщин с нарушениями сна и 33 436 женщин без (за исключением апноэ во сне), после чего была изучена связь между сомнологическими расстройствами и бесплодием (неспособность зачать ребенка в течение 1 года) [35]. У участниц с нарушением сна риск бесплодия был в 3,7 раза выше, чем у женщин группы сравнения; этот результат прослеживался на протяжении 11 лет наблюдения. Другое проспективное когортное исследование с участием 6873 специалистов по планированию беременности в Северной Америке показало, что плохое качество сна, которое оценили с помощью опросников, было связано с более длительным сроком до наступления беременности [36]. Также наблюдалась слабая обратная корреляция между короткой продолжительностью сна и количеством родов, а сменная работа не была связана с рождаемостью.

Суррогатным биомаркером метаболизма мелатонина является 6-сульфатоксимелатонин, метаболит гормона, экскретируемый с мочой. Он используется для оценки мелатонинового статуса среди пациенток с различными гинекологическими заболеваниями [37]. Снижение уровня 6-сульфатоксимелатонина в моче, связанное с более высокими уровнями эстрадиола и прогестерона, было обнаружено у женщин, работающих в ночную смену, по сравнению с женщинами, выходящими в дневное время [38]. При этом R. Luboshitzky и соавт. обнаружили повышенный уровень 6-сульфатоксимелатонина в моче в группе женщин с диагнозом синдром поликистозных яичников по сравнению с контролем, что авторы связывают с влиянием андрогенов при данной нозологии [39].

Клиническая значимость наших результатов является высокой, так как полученные данные позволяют планировать более крупные будущие исследования для поиска эффективных и патогенетически обоснованных методов коррекции метаболических нарушений и менструальной дисфункции у пациенток репродуктивного возраста. Корреляция мелатонина в слюне и его метаболита в суточной моче позволяет использовать метод определения гормона в слюне в качестве быстрого и информативного теста для уточнения мелатонинового статуса женщины.

Ограничениями нашего исследования могут быть условия проведения, так как набор участниц проводился только в федеральном научном центре, выявление нарушений сна только на основании анкетирования, без использования объективных методов диагностики (сомнография).

Нормальный циркадианный ритм имеет важное значение для энергетического гомеостаза организма. Появляется все больше работ, подтверждающих положительное влияние дотации экзогенного мелатонина на пациенток с ожирения. Тем не менее механизмы, лежащие в основе этого эффекта, еще до конца не изучены. Возможно, антиоксидантная и противовоспалительная функции этого гормона определяют его эффективность в коррекции метаболических нарушений. Несомненно, необходимы более крупные исследования, которые позволят осветить весь механизм действия мелатонина у пациенток с ожирением и, возможно, использовать его как терапевтическое средство для профилактики избыточного веса и его осложнений, а также установить роль сомнологических нарушений в развитии неблагоприятных репродуктивных исходов.

## ЗАКЛЮЧЕНИЕ

Качество и продолжительность сна могут иметь важное значение для репродуктивной функции женщины. Механизмы, лежащие в основе этой связи, вероятно, сложные и многофакторные. Нарушения циркадианного ритма могут влиять на овариальную функцию из-за сбоев в регуляции оси гипоталамус-эпифиз-яичники, в частности, за счет дефицита мелатонина и, как следствие, развившихся метаболических нарушений (резистентность к инсулину, окислительный стресс и системное воспаление). Мелатонин является потенциальным кандидатом для лечения ожирения за счет своего влияния на метаболизм инсулина и жировой ткани, липолиз и митохондриальную функцию. Гормон снижает дисфункцию жировой ткани благодаря своим антиоксидантным и противовоспалительным эффектам. Доказательства его защитных свойств и отсутствие значимых побочных реакций могут способствовать его продвижению в качестве терапевтического средства для пациентов с ожирением.

## ДОПОЛНИТЕЛЬНАЯ ИНФОРМАЦИЯ

Источник финансирования. Работа выполнена в рамках Государственного задания «Центральные и периферические патофизиологические механизмы развития болезней жировой ткани с учетом клинических и гормональных характеристик», 2020–2022 годы.

Конфликт интересов. Авторы декларируют отсутствие явных и потенциальных конфликтов интересов, связанных с публикацией настоящей статьи.

Участие авторов. Все авторы одобрили финальную версию статьи перед публикацией, выразили согласие нести ответственность за все аспекты работы, подразумевающую надлежащее изучение и решение вопросов, связанных с точностью или добросовестностью любой части работы.
